# Potential of ultra-high-resolution photon-counting CT of bone metastases: initial experiences in breast cancer patients

**DOI:** 10.1038/s41523-020-00207-3

**Published:** 2021-01-04

**Authors:** E. Wehrse, S. Sawall, L. Klein, P. Glemser, S. Delorme, H.-P. Schlemmer, M. Kachelrieß, M. Uhrig, C. H. Ziener, L. T. Rotkopf

**Affiliations:** 1 Division of Radiology, German Cancer Research Center, Heidelberg, Germany; 2 Medical Faculty, Ruprecht-Karls-University Heidelberg, Heidelberg, Germany; 3 Division of X-Ray Imaging and Computed Tomography, German Cancer Research Center, Heidelberg, Germany

**Keywords:** Cancer imaging, Preclinical research

## Abstract

Conventional CT scanners use energy-integrating detectors (EIDs). Photon-counting detector (PCD) computed tomography (CT) utilizes a CT detector technology based on smaller detector pixels capable of counting single photons and in addition discriminating their energy. Goal of this study was to explore the potential of higher spatial resolution for imaging of bone metastases. Four female patients with histologically confirmed breast cancer and bone metastases were included between July and October 2019. All patients underwent conventional EID CT scans followed by a high resolution non-contrast experimental PCD CT scan. Ultra-high resolution (UHR) reconstruction kernels were used to reconstruct axial slices with voxel sizes of 0.3 mm × 0.3 mm (inplane) × 1 mm (*z*-direction). Four radiologists blinded for patient identity assessed the images and compared the quality to conventional CT using a qualitative Likert scale. In this case series, we present images of bone metastases in breast cancer patients using an experimental PCD CT scanner and ultra-high-resolution kernels. A tendency to both a smaller inter-reader variability in the structural assessment of lesion sizes and in the readers’ opinion to an improved visualization of lesion margins and content was observed. In conclusion, while further studies are warranted, PCD CT has a high potential for therapy monitoring in breast cancer.

## Introduction

Conventional computed tomography (CT) scanner detectors consist of distinct detector elements^1^, with each of these elements hosting a scintillator that converts the incoming x-ray photon’s energy into visible light and a photodiode for the light registration. The generated and registered electrical signal corresponds to the total amount of absorbed and converted energy regardless of the number and individual energy of the incoming x-ray photons. Accordingly, the measured signal corresponds to the integrated, i.e., total amount of x-ray photon energy deposited in the detector volume in the specific intervals of each subsequent measurement.

Photon-counting detectors (PCDs), on the other hand, consist of a single layer semiconductor diode without the use of a dedicated scintillator^1^, directly converting a photon’s energy into a measurable charge cloud, corresponding to the photon’s energy. As the resulted signal is in the range of tens of nanoseconds^2,3^, small-sized detector pixels and advanced read-out electronics can be constructed to successfully discriminate photons reaching the detector almost simultaneously at high clinical x-ray flux rates^4,5^.

Photon-counting computed tomography (PCD CT) is therefore capable of counting single x-ray photons and discriminating them according to their energy^6–9^. Measurement of photon energy might especially be beneficial if it is combined with potential novel contrast agents consisting of medium-to-high atomic number elements, such as iodine, gadolinium, ytterbium, and bismuth^7,8,10,11^. These are currently being investigated and may allow for single-acquisition multi-phase imaging^12,13^.

Because of the aforementioned small pixels, current PCD CT scanners achieve a higher in-plane and longitudinal spatial resolution^4^ than conventional clinical energy-integrating detector (EID) CT, namely, up to 150 µm^14^. In combination with large image matrix reconstructions, improvements in the detection and visualization of higher-order bronchi^15^ and small pulmonary vessels^14^ as well as of the human inner ear and temporal bone^16,17^ are reported, whereas experience in clinical benefits in oncological imaging is still pending.

Breast cancer, the most common cancer among women, has a high incidence of bone metastases (BM), which is the most common distant form of metastasis^18–20^. They cause significant morbidity by fractures, hypercalcemia, and spinal cord compression as well as considerable mortality^21^, with mortality hazard ratios of five to six when compared to breast cancer patients without BM^22^.

Treatment of BM is of utmost importance, as skeletal complications can determine patients’ outcome and quality of life^22,23^. For response assessment of BM, several imaging modalities are available^24,25^. In planar bone scanning, ^99m^Tc-diphosphonate bone scanning, and positron emission tomography (PET) alone, most BMs cannot be assessed^25^. Hybrid imaging techniques, such as single-photon emission computed tomography/CT, PET/CT, and PET/magnetic resonance imaging (MRI) with bone-specific tracers like ^18^F-NaF or tumor-specific tracers like ^18^F-FDG achieve higher accuracies^26^. However, these modalities are more costly and are less widely available. Whole-body MRI acquisitions with diffusion weighted imaging and supporting T1- and T2-weighted sequences are also being investigated^27^ and provide valuable information about fat content and hypercellularity. These imaging modalities have acquisition times of 30–45 min^28^ and reveal millimetric resolution^29^. Because PCDs have been developed for clinical flux rates, the examination times for PCD CT do not differ significantly from EID CT, e.g., in the order of minutes for the whole procedure including positioning for whole-body acquisitions with submillimeter resolution. For osteoplastic and osteolytic bone lesions, the resolution improvement achieved with PCD CT can potentially be used to better differentiate between real tumor growth and therapy-associated sclerotic changes, i.e., pseudoprogression, as PCD CT reaches resolutions comparable with bone trabecular diameters^30^ and high-resolution images may allow for the detection of metastatic changes in shorter intervals.

After installation of a prototype PCD CT scanner in our institution, we are conducting an ongoing pilot study to gain insight in the potential advantages of this technique in various organs and diseases, intendedly exploiting its potential regarding resolution and signal-to-noise ratio. From this cohort, we extracted a subset of patients with advanced metastasized breast cancer to investigate the potential advantages of PCD CT imaging in assessing BMs. Examinations were performed on an experimental whole-body PCD CT (SOMATOM CounT), which is based on a SOMATOM Definition Flash dual-source CT scanner housing a prototype PCD and a conventional EID (Siemens Healthineers, Germany) that exclusively serves to obtain data from outside the limited field of view (FOV) covered by the PCD.

## Results

### Population characteristics

Four female patients diagnosed with late-stage osteoplastic metastasized breast cancer were examined, median age 61 years (45–68 years). The initial diagnosis was 14.5 years before the examination (2–23 years). Detailed grading and histological analysis results at the time of initial diagnosis are listed in Table 1.

**Table 1:** Reader’s opinion on different features in EID and PCD CT images.

Item	Reader’s opinion		
	Improved to EID CT	Neutral	Inferior to EID CT
Pat. 1, Osteolytic lesion (see Fig. 1a–e)			
Visualization of the lesion’s margin	4	0	0
Visualization of the lesion’s content (*)	4	0	0
Assessment of iliac cortical bone	3	1	0
Pat. 2, Osteoplastic lesion (see Fig. 2) and of PC and EID CT image stack			
Visualization of the lesion’s architecture	4	0	0
Assessment of the smallest focal lesions in the whole-image stack	4	0	0
Overview of tumor load and distribution in PC and EID CT image stack	3	1	0
Pat. 3, Fifth lumbar vertebra (see Fig. 3a, b)			
Discrimination of trabeculae from each other	4	0	0
Assessment of ventral formation of osteophytes	4	0	0
Pat. 4, Osteoplastic metastasis (red arrow in Fig. 3d) and the left iliac bone			
Visualization of the lesion’s margin	4	0	0
Assessment of Iliac spongiosa	3	1	0

### Patient 1

#### Case history

**Fig. 1:**
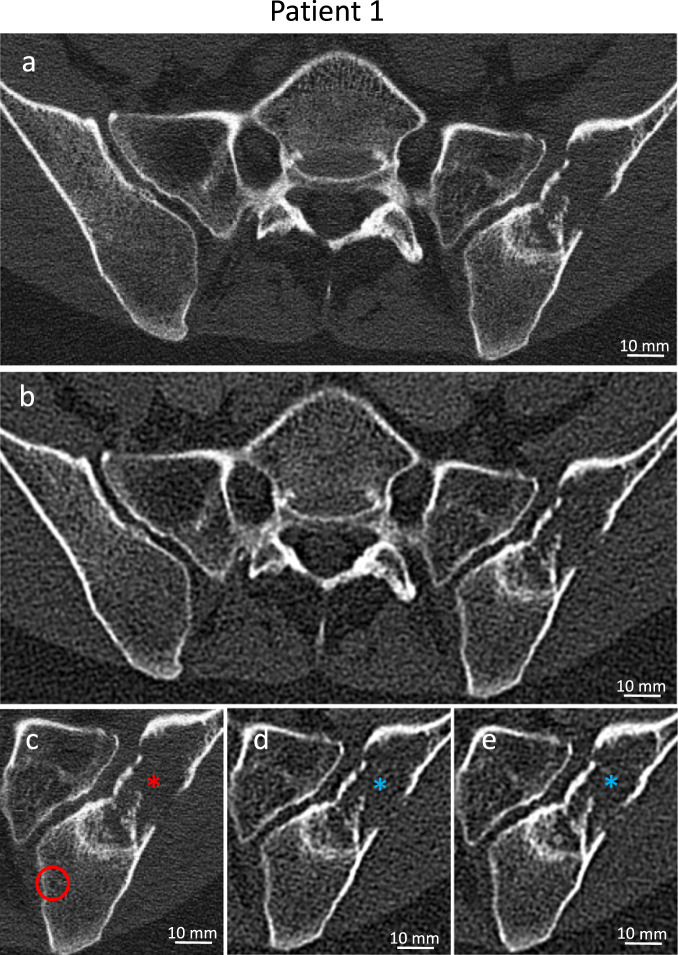
Examples of PCD CT and conventional EID CT images. **a**–**e** Unenhanced CT images of a 45-year-old patient with high-grade primary small cell carcinoma of the breast demonstrating an osteolytic lesion of the left iliac bone after radiation. **a**, **c** PCD CT images (U80f, 300 mAs, 2019/10, FOV 275 mm), **b**, **d**, **e** EID CT images (B80f, 124 mAs, **b**, **d** 2019/09, **e** 2019/12, FOV 500 mm). **c**, **d** are zoomed-in displays of **a** and **b**; **c**–**e** the small osseous defect shown in the PCD CT images (red circle) is visible to a moderate extent just in the low energy dataset (100 kV) of the dual-energy EID CT data.

The patient was first diagnosed in spring 2017 at the age of 42 years with a high-grade primary small cell carcinoma of the right breast, a subtype of the rare neuroendocrine breast cancer^31^. At diagnosis, the tumor had spread to the lymph nodes and exhibited both high estrogen receptor (ER) expression as well as high Ki-67 expression (Table 2). The patient received neoadjuvant chemotherapy consisting of etoposide and cisplatin before undergoing a modified radical mastectomy followed by radiation therapy of the resection bed and axilla 7 months after initial diagnosis. In summer 2019, she complained of increasing pain in the pelvis when receiving a routine ultrasound exam of the breast in our center. An abdominal CT demonstrated a large osteolytic BM in the left iliac bone, which was confirmed by a local biopsy. The lesion was irradiated in the following month and the patient remained on the postoperative regimen of letrozole and goserelin and was started on zoledronic acid. Eighteen months after initial diagnosis, the patient underwent both conventional CT and a PCD CT scan focused on the iliac BM in a span of 2 weeks (Fig. 1a–d). Three months later, another CT staging was performed (Fig. 1e). Since then, therapy and diagnostic follow-up has been continuing unchanged except for a 3-month course of leuprorelinacetate.

**Table 2:** Patient and tumor characteristics at the time of initial diagnosis.

Pat.	Disease	ER status	PR status	Her2neu status	Ki-67	T	N	M	L	G	Chemotherapy schemes at the time of image acquisition
1	Primary small cell carcinoma	Pos.	Pos.	Neg.	90%	cT3	cN+	–	–	G3	Goserelin + letrozole
2	ILC	Pos.	Pos.	Pos.	5–10%	pT2	pN0	M0	–	G2	Capecitabine
3	IDC	Pos.	Pos.	Neg.	25%	pT1c	pN0	–	–		Liposomal doxorubicin
4	NST	Pos.	Pos.	Neg.	20%	pT1c	pN0	–	L0	G2	Liposomal doxorubicin

*ILC* invasive lobular carcinoma, *IDC* invasive ductal carcinoma, *NST* no special type carcinoma.

#### Imaging discussion

Comparing conventional CT images of the osteolytic bone lesion (asterisk (*) in Fig. 1c–e) with PCD CT images demonstrates the benefit of higher spatial resolution in assessing structural bone changes and trabecular disruption. Comparing the dorsal borders of the lesion, PCD CT allows a clear depiction of trabecular lysis, while conventional CT shows only blurred border structures. In addition, the loss of trabecular density in the left ala of the sacrum can be clearly seen on PCD CT. The visualization of the lesion’s content and margin was improved in PCD CT images in comparison to EID CT images according the reader’s opinion (Table 1). Minor alterations of the iliac spongiosa, such as the small osseous defect in the medial iliac bone (red circle in the PCD CT image Fig. 1c) are hardly visible in EID CT images due to the noise grain size.

### Patient 2

The patient was first diagnosed with unifocal, medium-grade breast cancer in autumn 1996 at the age of 45 years. Histological staining revealed ER and progesterone receptor expression of immune reactive score 4 and 6, respectively (Table 2). Due to the relatively young age, the low hormone receptor expression, and a small tumor size, a cyclophosphamide, methotrexate, and 5-fluorouracil scheme was started after breast-conserving surgery and corresponding radiation therapy. Fifteen years later, multiple subsolid lung nodules were discovered and histopathologically confirmed to be metastases of the original tumor. The patient was subsequently randomized to receive either fulvestrant or fulvestrant combined with ribociclib (blinded). Over the next 2 years, she developed local recurrence and osteoplastic BMs mainly to the lumbar spine and pelvis. Following, she received repeated radiotherapy of the right axilla and medical treatment was changed to letrozole, zolendronic acid, and finally to palliative oral capecitabine. In July 2019, a routine EI CT staging was performed, followed by PCD CT examination 1 week later.

#### Imaging discussion

**Fig. 2:**
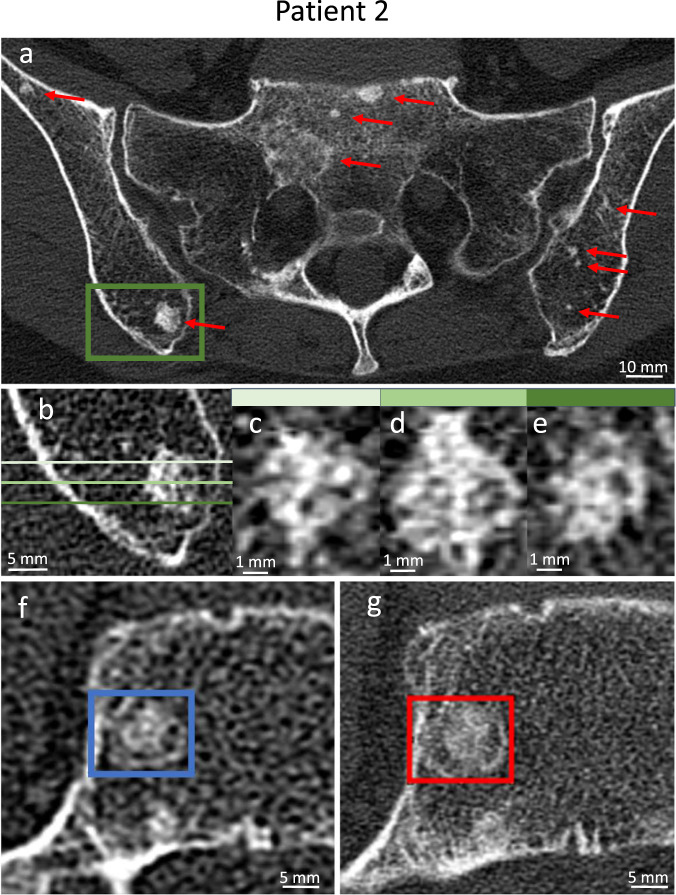
Unenhanced PCD and EID CT images of a 68-year-old patient with unifocal, medium-grade breast cancer and histologically confirmed osseous metastases. **a**–**e**, **g** PCD CT images (300 mAs, 2019/07), **f** EID CT image (B80f, 106 mAs, 2019/07). **a** Multiple osteoplastic metastases (red arrows) as small as 1 mm can be examined in detail (U80f reconstruction kernel, 1 mm slice thickness), **b**–**e** additional coronal reformations (**c**–**e**) at different positions (**b**) visualize the structure of a complex metastases (U70f, 0.25 mm slice thickness in *z*-axis). **f**, **g** Bone trabeculae of the fifth lumbar vertebra and a metastasis with sclerotic border (colored frames) can be adequately assessed in the PCD CT image (UHR reconstruction kernel U80f). CT window for all images [*C* = 500 HU, *W* = 1500 HU].

As can be seen in Fig. 2a, the patient had multiple disseminated osteoplastic bone lesions of varying size. Due to the relatively coarse trabecular structure lateral to the sacroiliac joint, differentiation of small metastases and benign focal changes is compromised due to partial volume effects. PCD CT allows an accurate depiction even of small focal lesions and their content (Fig. 2b–e, g). Figure 2f, g show an osteoplastic lesion in the fifth lumbar vertebra. An inner core and an outer sclerotic border can clearly be discriminated in both CT images, while the characterization of the tissue in between is improved in PCD CT images, according to the readers’ opinion (Table 1).

### Patient 3

#### Case history

The female patient was originally diagnosed with unifocal, low-grade breast cancer of the right breast in 2004 at the age of 50 years (Table 2). Following breast-conserving therapy and adjuvant radiation therapy, the patient was treated with letrozole and tamoxifen. Five years later, a ductal carcinoma in situ of the left breast was removed following the same procedure. Eleven years after initial diagnosis, breast cancer metastases were found in the right axilla.

CT staging showed disseminated osteoplastic BMs. Over the next 3 years, she received different regimens, including letrozole, palbociclib, vinorelbine, 5-flourouracil, fulvestrant, and ribociclib. In addition, she underwent external radiation of the frontal bone and of a singular hepatic metastasis. Forty-three months after initial diagnosis of the osseous lesions, the patient underwent conventional CT staging followed by a PCD CT of the lumbar spine a month later.

#### Imaging discussion

**Fig. 3:**
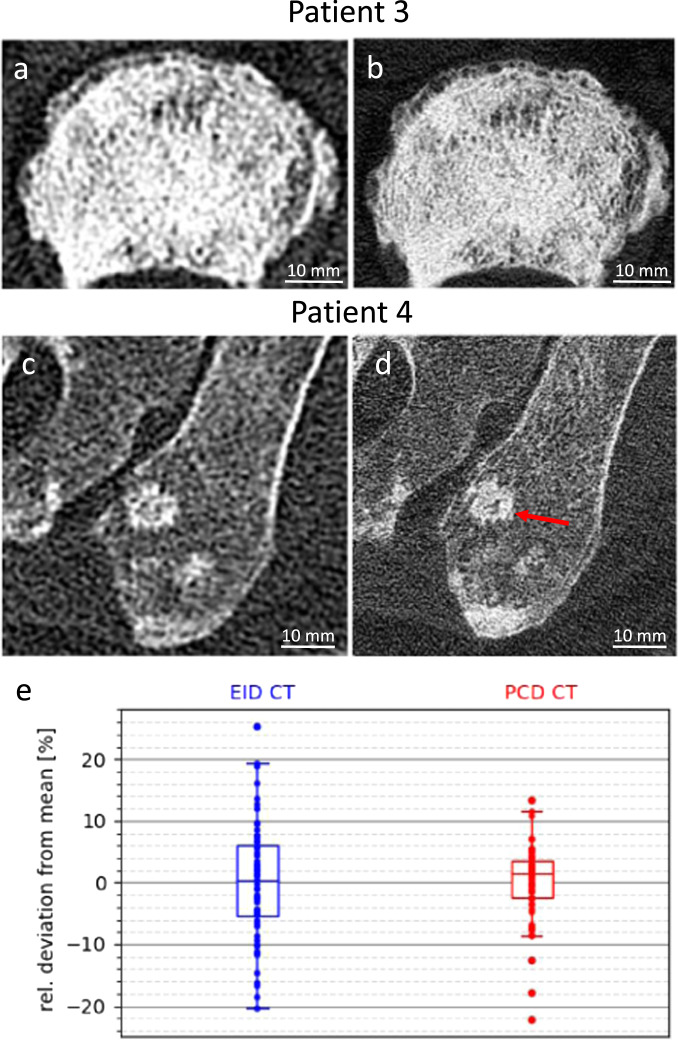
Examples of PCD CT and conventional EID CT images (top and central panel) and relative lesion area determined by the four readers (bottom panel). **a**, **b** Osteoplastic metastasis in the fifth lumbar vertebra of a 65-year-old patient. **a** EID CT image (2019/07, 102 mAs, B80f), **b** PCD CT image (2019/08, 300 mAs, U80f). **c**, **d** Osteoplastic metastases in the left iliac bone of a 57-year-old patient. **c** EI CT image (2019/12, 201 mAs, B80f), **d** PCD CT image (2019/09, 300 mAs, U80f), FOV: 500 mm for **a**, **c** and 275 mm for **b**, **d**. CT window for all images [*C* = 500 HU, *W* = 1500 HU]. **e** Relative lesion area (in percent from mean) and RMSE determined by the four readers in EID CT images (Blue) and PCD CT images (Red). Box plots demonstrating the inter-reader variability for all 15 lesions in EI and PCD CT (center line, median; box limits, upper and lower quartiles; whiskers, 1.5× interquartile range). Most lesions show a decreased inter-reader variability in PCD CT.

In contrast to the previous patient, Fig. 3a, b demonstrates an almost complete infiltration of the fifth lumbar vertebral body by osteoplastic metastases leading to coarsening of the vertebral trabeculae. At the ventral border of the vertebral body, a single row of degenerative osseous outgrowth is visible. Assessment on image features is listed in Table 1. Again, the possibility of visualizing single trabeculae may allow a more accurate assessment of the metastatic process.

### Patient 4

#### Case history

The patient was initially diagnosed with unifocal hormone receptor-positive breast cancer of the right breast in summer 2005 at the age of 43 years (Table 2). After a breast-sparing mastectomy, radiation therapy, and adjuvant antihormonal therapy, the patient remained symptom-free until 2015, when she noticed increasing pain along the whole spine. A CT scan showed disseminated osseous metastases in the whole axial skeleton as well as a suspicious lung nodule, which turned out to be a metastasis of the before-mentioned breast cancer. Due to the advanced disease, palliative therapy including radiotherapy of several vertebrae and ribs and letrozole was started. Varying chemotherapy schemata followed, which failed to significantly inhibit tumor growth. Due to an increasing hepatic tumor load, the general condition worsened. Sadly the patient passed away in December 2019, 14 years after the initial cancer diagnosis.

#### Imaging discussion

In Fig. 3c, d, several osteoplastic metastases of varying size and structure along the left iliosacral joint are depicted. In the PCD CT image, it is clearly visible that the tumor spiculae of the central metastasis in the medial margin are oriented in parallel to the trabecular structure. According to the readers’ opinion, the fine structure of the iliac spongiosa is better assessable in the PCD CT images (Table 1).

#### Inter-reader variability in the assessment of lesion sizes

Lesion sizes show relative deviations from the mean in the order of 20%. In 10 out of 15 total lesions (66.7%), the root-mean-squared error (RMSE) was lower in the PCD CT images, as demonstrated in Fig. 3e.

## Discussion

This case series illustrates the value of PCD detectors in assessing bone lesions in metastasized breast cancer. This detector allows, at the same dose, taking images with significantly higher spatial resolution^4^. Among others, this is reflected by smaller noise grain sizes. Using PCD CT high-resolution kernels, overshoots and undershoots often observed at sharp edges in EID CT using sharp kernels is reduced, resulting in a more subtle image impression that is apparently favored by the majority of the readers. Tumor margins can be depicted much clearer and assessment of the structural integrity of remaining trabecular bone becomes easier. As the remaining components including the x-ray tubes of this PCD CT scanner are identical to standard clinical CT scanners, risk of technical failure is kept at a minimum, leaving essentially no significant downsides to upgrading the detector.

Without nuclear medicine techniques, assessing the response of BMs to treatment relies mainly on morphological criteria. For example, the MD Anderson bone response criteria^32^ assign complete response only when the previously osteolytic lesion shows complete sclerotic fill-in, while partial response is assigned in cases of only incomplete fill-in or development of a sclerotic rim. It is therefore vital to depict structural bone changes even in small lesions, a task that remains challenging for lesions that do not exceed the standard voxel size by at least a magnitude. Still, while several studies have investigated the use of PCD for clinical applications^10,15,33–35^, larger clinical studies utilizing PCD are required before widespread adoption becomes possible.

This study has limitations. The examination protocol was adapted from high-resolution EID CT protocols used for temporal bone and inner ear imaging^36,37^ and is as such not directly transferable to whole-body examinations. Since the focus of this study was not a direct comparison with EID CT but to explore new potential clinical applications, dose levels were not matched to clinical EID CT levels. A recently published study focusing on sinus and temporal bone examination utilized comparable doses^17^. The current experimental implementation of the PCD into an existing dual-source EID CT scanner suffers from technical limitations, which are no general limitation of the PCD technology itself. Among others, the axial FOV and also the *z*-coverage, with the latter resulting in slower scan speeds, are limited. Furthermore, the available image reconstruction and post-processing options are not yet fully available and the detector elements are currently read out in groups of 2 × 2 pixels, due to restricted data transfer rates^4^.

Quantitative material images, such as calcium images and virtual non-calcium maps, would allow for an improved visualization^38^, assessment of the lesions’ content, and a precise quantification of therapy results. For longitudinal studies, the calcium density has to be corrected to a varying fat portion in the bone marrow^39^, which is possible by dual-energy CT^40^. As theoretically predicted^41^ and experimentally shown^42,43^, single-source PCD CT is inferior at least to the latest dual-source EID CT systems regarding spectral separation, due to different x-ray source spectra and prefiltration in the latter. Additionally, current PCD CT systems are compromised by spectral distortions and non-ideal physical effects^44^. Combining PCDs and technologies such as dual-source CTs may therefore lead to additional improvements and may facilitate further dose reductions.

In parallel with technical modifications, clinical investigations have to focus on several questions. In future follow-up examinations, morphological and spectral differences between bone lesions with vital metastases and reactive sclerosis due to systemic therapy or radiotherapy have to be investigated systematically.

In conclusion, while research into potential clinical applications is ongoing, and key applications have yet to be identified, the higher resolution and improved image quality of PCD CT images potentially offers benefits for the assessment of BMs of breast cancer.

## Methods

### Study design

This prospective case series was approved both by the Ethics Committee of the Medical Faculty of Heidelberg according to the Declaration of Helsinki of 2013 and the Federal Office for Radiation Protection, and all patients gave their informed consent (German Clinical Trials Register DRKS00017759). Candidates for PCD CT imaging were selected from patients undergoing regular oncological follow-up examinations in our clinic with Eastern Cooperative Oncology Group performance status^45^ of 0–2, histologically confirmed breast cancer, and BMs in the pelvis or lumbar spine between July and October 2019. For comparison, we used clinical routine EID CT images acquired before and after the PCD CT exams. Lesions previously detected in these EID CT scans were selected as target lesions, which were placed in the center of the scan volume in the PCD CT scans.

### CT imaging and image analysis

All patients underwent routine contrast-enhanced CT staging at a SOMATOM Definition Flash (Siemens Healthineers, Germany) dual-source CT scanner operating either in single-energy mode (one patient) or dual-energy mode (three patients), as listed in Table 3. Tube current time products per source ranged from 90 to 235 mAs. For dual-energy scans, both detector signals were averaged for improving signal-to-noise ratios. PCD CT acquisitions were performed with 300 mAs using the ultra-high-resolution mode of the PCD (0.25 mm pixel size in the isocenter). Additionally, a data completion scan (120 kV, 30 mAs, 25 mGycm, CTDI of 2.1 mGy) was done to avoid truncation artifacts^46^ caused by the limited field of measurement of the experimental PC detector. For axial images, reconstruction was performed using a routine B80f kernel for conventional CT and a high-resolution U80f kernel for PCD CT and pixel size of 0.3 × 0.3 mm with 1 mm slice thickness. The maximal scan length was fixed at 10 cm in craniocaudal direction.

**Table 3:** Acquisition and reconstruction parameters for this study (if not mentioned otherwise).

Scan mode	EID	PCD
Scan type	Spiral	Sequential
Rotation time	0.5 s	1.0 s
Collimation	32 × 0.6	32 × 0.25
Pitch	0.6	–
Tube voltage	100 kV/140 kV + Sn or 100 kV	120 kV
Effective tube current	90–235 mAs per source	300 mAs
CARE DOSE 4D	ON	OFF
CTDI	7.0–11.4 mGy	24.3 mGy
Recon algorithm	wFBP	wFBP
Recon kernel	B80f	U80f
Slice thickness	1 mm	1 mm
Slice increment	0.5 mm	0.5 mm
In-plane pixel size	0.3 mm × 0.3 mm	0.3 mm × 0.3 mm
Display window	[*C* = 500 HU, *W* = 1500 HU]	[*C* = 500 HU, *W* = 1500 HU]

### Reader study

Two board-certified radiologists (9 and 8 years of experience in oncologic imaging) and two residents (1 year experience in oncologic imaging) assessed volumetric image stacks of PCD CT and conventional CT on a standard clinical reading console with patient identity blinded and in randomized order. Due to differences in FOV, acquisition parameters, and spatial resolution, the CT modality could not be blinded. Viewing parameters were freely adjustable and images in axial, coronal, and sagittal reformation were provided. Characteristic image sections were rated with respect to specific parameters (detailed below) and graded on a three-point Likert scale. The results of the survey are demonstrated next to the image section. Additionally, the readers were prompted to determine the area of 15 focal osteoplastic lesions (10 lesions from Pat. 2 and 5 lesions from Pat. 4, sizes ranging from 30 to 1500 mm^2^) by measuring the long and short axes as per RECIST guidelines^47^.

### Statistics

All statistical analyses were performed using Python 3.8 (Python Software Foundation). Parameters were compared as absolute quantities and relative percentage points. Due to the low number of both images and readers, no significance testing was done.

For the inter-reader variability of the different lesion sizes, we calculated the relative RMSE according to ${\rm{RMSE}} = \sqrt {\mathop {\sum }\nolimits_{n = 1}^4 \frac{{\left( {s_n - \bar s} \right)^2}}{4}}$ with *s*_*n*_ as the lesion area and $\bar s$ as the mean lesion area over all readers.

### Reporting summary

Further information on research design is available in the Nature Research Reporting Summary linked to this article.

## Supplementary information

Reporting Summary Checklist

## Data Availability

The data generated and analyzed during this study are described in the following data record: 10.6084/m9.figshare.13186661^48^. All demonstrated CT images are available as part of the data record as pseudonymized DICOM files. The acquisition and reconstruction parameters are available (as metadata in each file) so any user will be able to adapt the CT window. These DICOM files underlie Figs. 1 and 2 and Table 3. The file names include the figures to which they apply. Additionally, the Excel spreadsheet npj_breast_cancer_PCCT_Reader-Study.xlsx, which is also part of the data record, contains the lesions diameters according to the four readers. All readers are co-authors on the related manuscript and allow the publication of their assessments. All data on the reader’s opinion on different features in EID and PCD CT images are presented in Table 1 of the related manuscript. The patient and tumor characteristics at the time of initial diagnosis, which are contained in Table 2 of the related manuscript, are data from given physicians’ letters, and so cannot be shared without jeopardizing patients’ anonymity. Any requests for access to this data should be made to the corresponding author.

## Code availability

All statistical analyses were performed using standard statistical libraries in Python 3.8 (Python Software Foundation). Code is available on reasonable request from any qualified researcher.
