# Supplementary material for: Potential of ultra-high-resolution photon-counting CT of bone metastases: initial experiences in breast cancer patients
Source: NPJ Breast Cancer. 2021 Jan 4;7:3. doi: 10.1038/s41523-020-00207-3 (PMC7782694; doi:10.1038/s41523-020-00207-3)
Supplement: Supplementary file 1 — Reporting Summary Checklist [file 41523_2020_207_MOESM1_ESM.pdf]

nature research

Corresponding author(s): Eckhard Wehrse

Last updated by author(s): Nov 8, 2020

Reporting Summary

Nature Research wishes to improve the reproducibility of the work that we publish. This form provides structure for consistency and transparency in reporting. For further information on Nature Research policies, see our Editorial Policies and the Editorial Policy Checklist.

Statistics

For all statistical analyses, confirm that the following items are present in the figure legend, table legend, main text, or Methods section.

n/a	Confirmed	
☐	☒	The exact sample size (*n*) for each experimental group/condition, given as a discrete number and unit of measurement
☐	☒	A statement on whether measurements were taken from distinct samples or whether the same sample was measured repeatedly
☐	☒	The statistical test(s) used AND whether they are one- or two-sided<br>*Only common tests should be described solely by name; describe more complex techniques in the Methods section.*
☐	☒	A description of all covariates tested
☐	☒	A description of any assumptions or corrections, such as tests of normality and adjustment for multiple comparisons
☐	☒	A full description of the statistical parameters including central tendency (e.g. means) or other basic estimates (e.g. regression coefficient) AND variation (e.g. standard deviation) or associated estimates of uncertainty (e.g. confidence intervals)
☐	☒	For null hypothesis testing, the test statistic (e.g. *F*, *t*, *r*) with confidence intervals, effect sizes, degrees of freedom and *P* value noted<br>*Give P values as exact values whenever suitable.*
☐	☒	For Bayesian analysis, information on the choice of priors and Markov chain Monte Carlo settings
☐	☒	For hierarchical and complex designs, identification of the appropriate level for tests and full reporting of outcomes
☐	☒	Estimates of effect sizes (e.g. Cohen's *d*, Pearson's *r*), indicating how they were calculated

Our web collection on statistics for biologists contains articles on many of the points above.

Software and code

Policy information about availability of computer code

Data collection	For reconstruction of the PCD CT images: ReconCT 14.0.1.45000, Dicom-Viewer for Figures: RadiAnt DICOM Viewer Version 2020.1.1<br>Reader Study: GE Healthcare Centricity PACS
Data analysis	Box Plots: Python 3.8 (Python Software Foundation) and

For manuscripts utilizing custom algorithms or software that are central to the research but not yet described in published literature, software must be made available to editors and reviewers. We strongly encourage code deposition in a community repository (e.g. GitHub). See the Nature Research guidelines for submitting code & software for further information.

Data

Policy information about availability of data

All manuscripts must include a data availability statement. This statement should provide the following information, where applicable:
- Accession codes, unique identifiers, or web links for publicly available datasets
- A list of figures that have associated raw data
- A description of any restrictions on data availability

Data is available on reasonable request from any qualified researcher.

Field-specific reporting

Please select the one below that is the best fit for your research. If you are not sure, read the appropriate sections before making your selection.

☒ Life sciences ☐ Behavioural & social sciences ☐ Ecological, evolutionary & environmental sciences

For a reference copy of the document with all sections, see nature.com/documents/nr-reporting-summary-flat.pdf

Life sciences study design

All studies must disclose on these points even when the disclosure is negative.

Sample size	For this explorative study, no sample size calculation was performed and we included four patients with histologically confirmed breast cancer and bone metastases between July and October 2019
Data exclusions	No data was excluded.
Replication	We presented the experimental Photon-Counting Detector CT, acquisition and reconstruction parameters in the paper. Although the demonstrated CT images represent acquisitions of individual probands, the texture and pattern or metastases is typical for patients in these advanced state of disease.
Randomization	For this explorative study of 4 patients no randomization of cohorts took place.
Blinding	We considered a blinding of the imaging modality for the reader study. However, due to different FOV and reconstruction kernels, one can differentiate PCD /EID CT at first sight.

Reporting for specific materials, systems and methods

We require information from authors about some types of materials, experimental systems and methods used in many studies. Here, indicate whether each material, system or method listed is relevant to your study. If you are not sure if a list item applies to your research, read the appropriate section before selecting a response.

Materials & experimental systems

n/a	Involved in the study	
☒	☐	Antibodies
☒	☐	Eukaryotic cell lines
☒	☐	Palaeontology and archaeology
☒	☐	Animals and other organisms
☐	☒	Human research participants
☐	☒	Clinical data
☒	☐	Dual use research of concern

Methods

n/a	Involved in the study	
☒	☐	ChIP-seq
☒	☐	Flow cytometry
☒	☐	MRI-based neuroimaging

Human research participants

Policy information about studies involving human research participants

Population characteristics	Four female patients with histologically confirmed breast cancer and bone metastases were included between July and October 2019, diagnosed with late stage osteoplastic metastasized breast cancer were examined, median age 61 y (45 to 68 y). The initial diagnosis was 14.5 y before the examination (2 to 23 y).
Recruitment	We recruited probands, with histologically confirmed breast cancer and bone metastases, who underwent regular EID CT scans at our department. All probands represent patients with advanced metastasized breast cancer, especially not at a stage of first diagnosis of osseus metastases.
Ethics oversight	Ethics Committee of the Medical Faculty of Heidelberg according to the Declaration of Helsinki of 2013 and the Federal Office for Radiation Protection

Note that full information on the approval of the study protocol must also be provided in the manuscript.

Clinical data

Policy information about clinical studies

All manuscripts should comply with the ICMJE guidelines for publication of clinical research and a completed CONSORT checklist must be included with all submissions.

Clinical trial registration	German Clinical Trials Register DRKS00017759
Study protocol	The study protocol is available at https://drks.de/drks_web/navigate.do?navigationId=trial.HTML&TRIAL_ID=DRKS00017759
Data collection	Candidates for PCD CT imaging were selected from patients undergoing regular oncological follow up examinations in our clinic with ECOG performance status of 0 to 2, histologically confirmed breast cancer and bone metastases in the pelvis or lumbar spine between July and October 2019. We included the patient in our study after informed consent.
Outcomes	This is an explorative radiological study. We compared the image quality of the PCD CT with clinical EID CT by a reader study of 4 radiologists. For the rebuttal letter we additionally investigated line curve profiles at sharp boundaries in detail.
